# Relationships between positive schemas and life satisfaction in psychiatric inpatients

**DOI:** 10.3389/fpsyg.2022.1061516

**Published:** 2022-12-15

**Authors:** Danni Chi, Haiyun Zhong, Yubo Wang, Hong Ma, Yuanyuan Zhang, Xiangju Du

**Affiliations:** Psychosomatic Medicine Department, Ningbo Kangning Hospital, Ningbo, China

**Keywords:** early adaptive schema, positive schema, life satisfaction, resilience, depression, anxiety

## Abstract

**Introduction:**

Unlike the extensively examined early maladaptive schemas, positive schemas have rarely been examined in psychiatric patients. This study aimed to investigate the relationships between anxiety, depression, life satisfaction, resilience, and positive schemas in psychiatric inpatients with generalized anxiety disorder or major depressive disorder and explore their associations. A sample of 140 psychiatric inpatients with anxiety and depression, aged between 18 and 72 years (*M_age_* = 35.62, *SD* = 13.87) participated in this study.

**Methods:**

A majority were women (*n* = 98, 70.0%). The analyses examined resilience and anxiety/depression as mediators between positive schema and life satisfaction.

**Results:**

Based on statistical analyses, resilience and anxiety/depression were parallel rather than serial mediators between positive schemas and life satisfaction. These results confirmed the positive association between positive schemas and life satisfaction, and identified mechanisms between them: directly associated and indirectly associated through the parallel mediations of resilience and anxiety/depression.

**Discussion:**

The findings of this study suggest that higher levels of positive schemas in psychiatric inpatients are associated with greater life satisfaction directly and through the mediation of reduced psychopathological emotions and increased resilience. Longitudinal studies are needed to explore the associations between positive schemas and negative and positive outcomes and the mechanisms underlying these associations in clinical populations.

## Introduction

Early schemas are broad and pervasive themes comprising emotions, cognitions, memories, bodily sensations, and neurobiological reactions regarding oneself and one’s relationship with others, which are developed in childhood or adolescence and might be maintained throughout one’s lifetime (Young et al., 2003). Individuals are likely to develop early adaptive schemas (EASs) or positive schemas and healthy and adaptive personal and social functioning if their core emotional needs are sufficiently met, otherwise early maladaptive schemas (EMSs) or negative schemas and maladaptive functioning may be developed (Young et al., 2003; Lockwood and Perris, 2012). Empirical studies confirm this claim. Positive schemas have explained additional variance in psychopathological outcomes, resilience, life satisfaction, and self-efficacy after controlling for EMSs in the general population (Louis et al., 2018; Paetsch et al., 2020). Thus, positive schemas and EMSs are different constructs rather than opposite ends of a continuum, and individuals with EMSs in some areas can also have positive schemas in other aspects.

EMSs have been investigated and confirmed as transdiagnostic psychopathological factors leading to affective dysfunction and personality disorders (Wang et al., 2010; Bamelis et al., 2014; Peeters et al., 2021). In contrast, the concept of positive schemas has been proposed for about a decade (Lockwood and Perris, 2012), and few studies have examined EASs or positive schemas in either community or psychiatric populations. A few studies have reported positive associations between positive schemas and resilience and life satisfaction, and negative associations with psychopathological outcomes in the general adult population (Louis et al., 2018; Paetsch et al., 2020). A study found similar results in a general sample of 172 adolescents (Keyfitz et al., 2013). However, it remains unclear how positive schemas and these factors are associated, especially in psychiatric patients. Investigating the mechanism of the associations between positive schemas, resilience, anxiety, depression, and life satisfaction will deepen the current understanding of the positive schema theory and provide possibilities for further exploration of its use in clinical settings.

This study proposed a serial mediation model to examine resilience and anxiety/depression as mediators between positive schemas and life satisfaction in psychiatric inpatients with anxiety/depression. Resilience refers to personal qualities that enable individuals to cope with and thrive during stressful events (Connor and Davidson, 2003). Studies have found that higher levels of resilience reported lower levels of anxiety and depression and greater life satisfaction in undergraduates and patients with depression (Yu and Zhang, 2007; Cheng et al., 2020). Moreover, in a cohort study, higher levels of resilience and lower levels of anxiety and depression predicted greater life satisfaction (Beutel et al., 2010). Thus, significant associations between positive schemas, resilience, anxiety, depression, and life satisfaction have been confirmed. It is possible that people with higher levels of positive schemas are also likely to be more resilient, experience lower levels of anxiety and depression, and thus, perceive greater life satisfaction. This study examines this possibility. More specifically, resilience and anxiety/depression were hypothesized as serial mediators between positive schemas and life satisfaction.

This study explored the following hypotheses:

*Hypothesis1*: Positive schema is associated with life satisfaction through the serial mediation of resilience and anxiety.

*Hypothesis 2*: Positive schema is associated with life satisfaction through the serial mediation of resilience and depression.

## Materials and methods

This study used a cross-sectional design to investigate the associations between positive schemas, resilience, anxiety, depression, and life satisfaction, and further explored the mechanism of these associations.

### Procedure

This study was completely anonymous and voluntary, and was approved by the Institutional Ethics Committee of Ningbo Kangning Hospital (NBKNYY-2021-LC-50). Recruitment lasted from 16 February, 2022 to 19 April, 2022. Recruiting posters were placed in a ward of mental health hospital; Patients in the ward were those whose main diagnosis was major depressive disorder (MDD) or generalized anxiety disorder (GAD) according to the fifth edition of the Diagnostic and Statistical Manual of Mental Disorder (American Psychiatric Association, 2013). Inpatients with GAD/MDD could scan the QR code from the posters to answer the survey on their first day in hospital. The inclusion criteria were: aged between 18 and 75 years, fluent in Chinese, and capable of understanding and responding to the survey.

### Participants

A total of 167 inpatients submitted signed informed consent to participate in the study. After screening for the inclusion criteria and outliers, a final sample of 140 inpatients was obtained. Among the 27 removed cases, 20 of them were removed as their ages were lower than 18, six were removed as the average respondent time per item was shorter than 2 s, and one case was removed due to missing data of more than 50%.

### Measurements

#### Demographics

Participants were asked to provide information on their age, sex, and educational background.

#### Positive schema

Positive schemas were measured using the Chinese version of the Young Positive Schema Questionnaire (YPSQ; Louis et al., 2018), which is a self-reporting inventory comprising 14 factors and 54 items (Louis et al., 2018). The participants were asked to rate the degree to which each of the statements fit them over the past month. Each item is rated on a 6-point Likert-type scale ranging from 1 (completely untrue of me) to 6 (describes me perfectly). Higher scores indicate more positive schemas. The Cronbach’s alpha of each factor in the Chinese YPSQ was above 0.70 in community samples (Louis et al., 2018; Chi et al., 2022). The Cronbach’s alpha of the total YPSQ score was 0.98 in this study.

#### Life satisfaction

The life satisfaction subscale of the comprehensive inventory of thriving (CIT; Su et al., 2014) was used to measure life satisfaction over the past month. The original CIT has been proven valid and reliable (Duan et al., 2020), and subscales have been suggested for selective use (Su et al., 2014). This self-reported life satisfaction subscale comprises three items. Each item is rated on a 5-point Likert-type scale ranging from 1 (strongly disagree) to 5 (strongly agree). Higher scores indicate greater life satisfaction. The Cronbach’s alpha of this scale was 0.96 in this study.

#### Resilience

The Chinese version of the 10-item Connor-Davidson Resilience Scale (CD-RISK-10; Connor and Davidson, 2003) was used to measure the resilience level of the participants over the past month. The CD-RISK-10 is a 5-point Likert-type scale ranging from 0 (not true at all) to 4 (true nearly all the time). Higher scores indicate greater resilience. The CD-RISK-10 is a self-report tool that has shown good validity and reliability among Chinese college students (Yu and Zhang, 2007; Cheng et al., 2020). Its Cronbach’s alpha was 0.96 in this study.

#### Anxiety

The 7-item Generalized Anxiety Disorder (GAD-7; Spitzer et al., 2006) was used to examine the anxiety level of the study participants, who were asked to rate their degree of anxiety over the previous month. Each item is rated on a 4-point Likert-type scale ranging from 0 (not at all) to 3 (nearly every day). Higher scores indicate higher levels of anxiety. The GAD-7 has good validity and reliability for anxiety screening in clinical practice and research. The Cronbach’s alpha of the GAD-7 in this study was 0.96.

#### Depression

The 9-item Patient Health Questionnaire (PHQ-9; Kroenke et al., 2001) is a self-administered questionnaire used to measure depressive mood. Participants were asked to rate their degree of depression over the previous month. Each item is rated on a 4-point Likert-type scale ranging from 0 (not at all) to 3 (nearly every day). Higher scores indicate higher levels of depression. The Cronbach’s alpha for the PHQ-9 in this study was 0.94.

### Statistical analyses

The data analyses included descriptive statistics, bivariate analysis, and hypothesis testing. Prior to this, missing data, normality, outliers, linearity, average response time, and multicollinearity were considered (Tabachnick and Fidell, 2007; Huang et al., 2012). No significant issues were identified in this study. The significance level was set at *p* < 0.05. Scale scores were computed as the mean of the items. Bootstrapped independent samples *t*-tests were used to assess the mean differences between sexes. Bivariate correlations of continuous variables were examined using Pearson’s correlation. Parallel and serial mediations were conducted using the SPSS plug-in PROCESS macro models 4 and 6, respectively (version 3.4; Hayes, 2013). An effect was considered significant if the confidence interval (CI) did not include zero.

## Results

### Descriptive statistics

Table 1 presents the demographics of the participants. Among the 140 participants, majority were women (*n* = 98, 70%), and more than half went to college or above (*n* = 90, 64.3%). In addition, the participants’ ages ranged from 18 to 72 years, with a mean of 35.62 years (*SD* = 13.87).

**Table 1:** Demographics of participants.

Demographics		Frequency	Percentage
**Age**			
	Mean (SD)	35.62 (13.87)	
	18–20	23	16.4
	21–30	36	25.7
	31–40	29	20.7
	41–50	26	18.6
	51–60	14	10.0
	61–70	6	4.3
	Above 70	1	0.7
	Missing	5	3.6
**Gender**			
	Men	42	30.0
	Women	98	70.0
	Trans gender	0	0
**Education level**			
	Middle school and below	26	18.6
	High school	24	17.1
	College or university	82	58.6
	Postgraduate and above	8	5.7

*n* = 140. SD, standard deviation.

The level of the overall positive schemas (*mean* = 4.14, *SD* = 1.03) was higher in the present study than among a Chinese community sample (Chi et al., 2022): *t* = −3.8, *p* < 0.001. No significant differences were identified between men and women in levels of positive schema (*t* = −0.53, *p* = 0.60), resilience (*t* = −0.21, *p* = 0.83), life satisfaction (*t* = 0.13, *p* = 0.90), anxiety (*t* = −0.25, *p* = 0.81), and depression (*t* = −1.18, *p* = 0.24).

### Bivariate correlations

As shown in Table 2, age was positively correlated with positive schema, resilience, and life satisfaction and negatively correlated with anxiety. Positive schema, resilience, and life satisfaction were positively correlated with one another, and all were negatively correlated with anxiety and depression. Thus, age was controlled for in the mediation analysis.

**Table 2:** Correlation analysis of continuous variables.

	1	2	3	4	5
1. Age					
2. Positive schema total	0.42**				
3. Resilience	0.27**	0.86**			
4. Life satisfaction	0.32**	0.77**	0.76**		
5. Anxiety	−0.18*	−0.46**	−0.47**	−0.54**	
6. Depression	−0.10	−0.46**	−0.47**	−0.53**	0.90**

*N* = 130–140. **p < 0.01 (2-tailed), *p < 0.05 (2-tailed).

### Hypotheses testing

**Figure 1:**
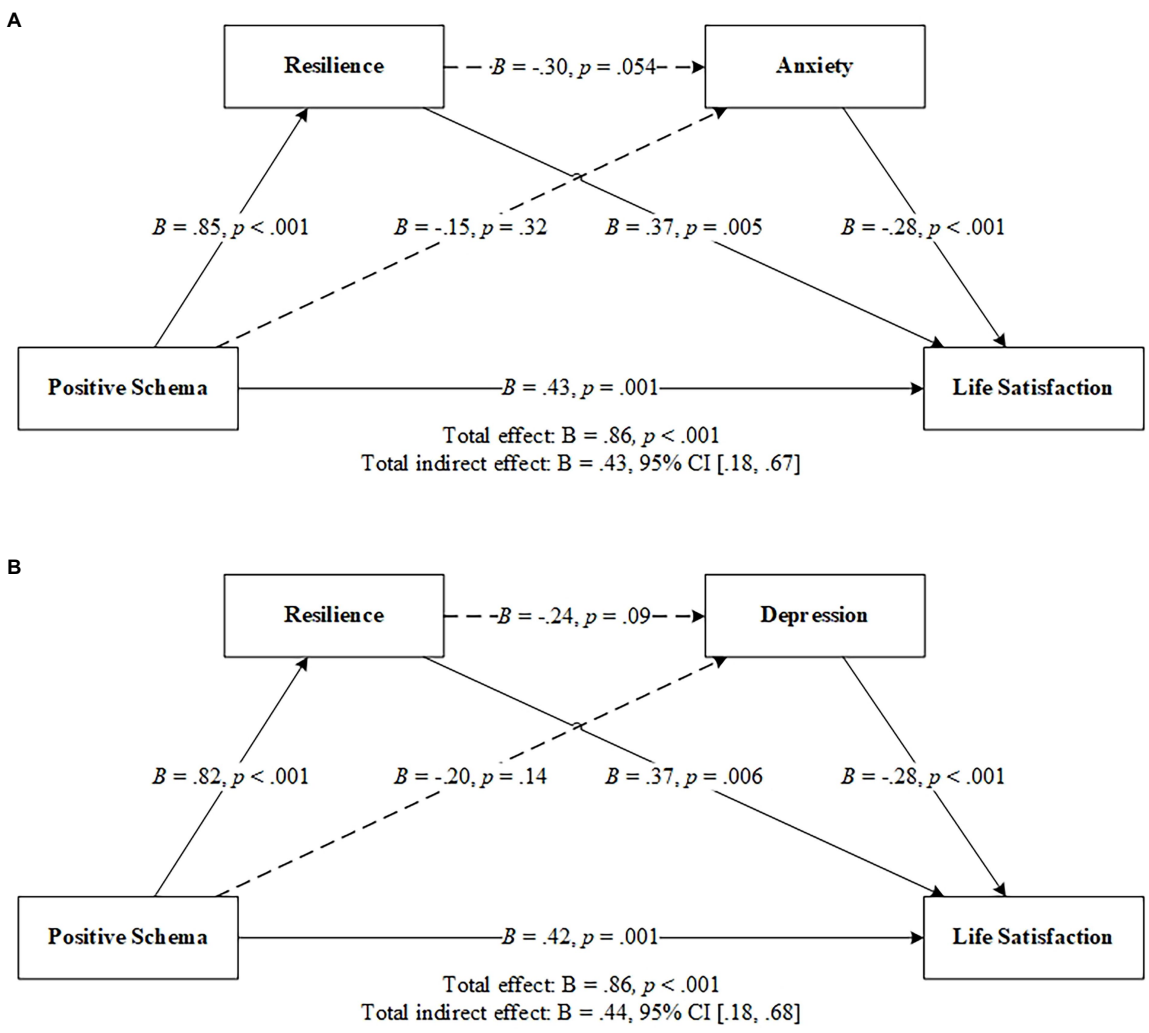
Resilience and anxiety **(A)**/depression **(B)** as serial mediators between positive schema and life satisfaction. *N* = 129. CI, confidence interval. Solid lines indicate significant paths, and dotted lines indicate nonsignificant paths. Results were based on 5,000 bootstrap samples.

Hypotheses 1 and 2 explored the roles of resilience and anxiety (Hypothesis 1) and depression (Hypothesis 2) as serial mediators between positive schemas and life satisfaction. However, neither hypothesis was supported, as the indirect effects of positive schema through resilience and anxiety/depression on life satisfaction were not significant (95% CI included zero, as shown in Supplementary Table S1). The indirect effects of positive schema on life satisfaction through anxiety/depression were also not significant (Figure 1; Supplementary Table S1). Notably, the indirect effects of positive schemas through resilience on life satisfaction were significant.

### Explorations

**Figure 2:**
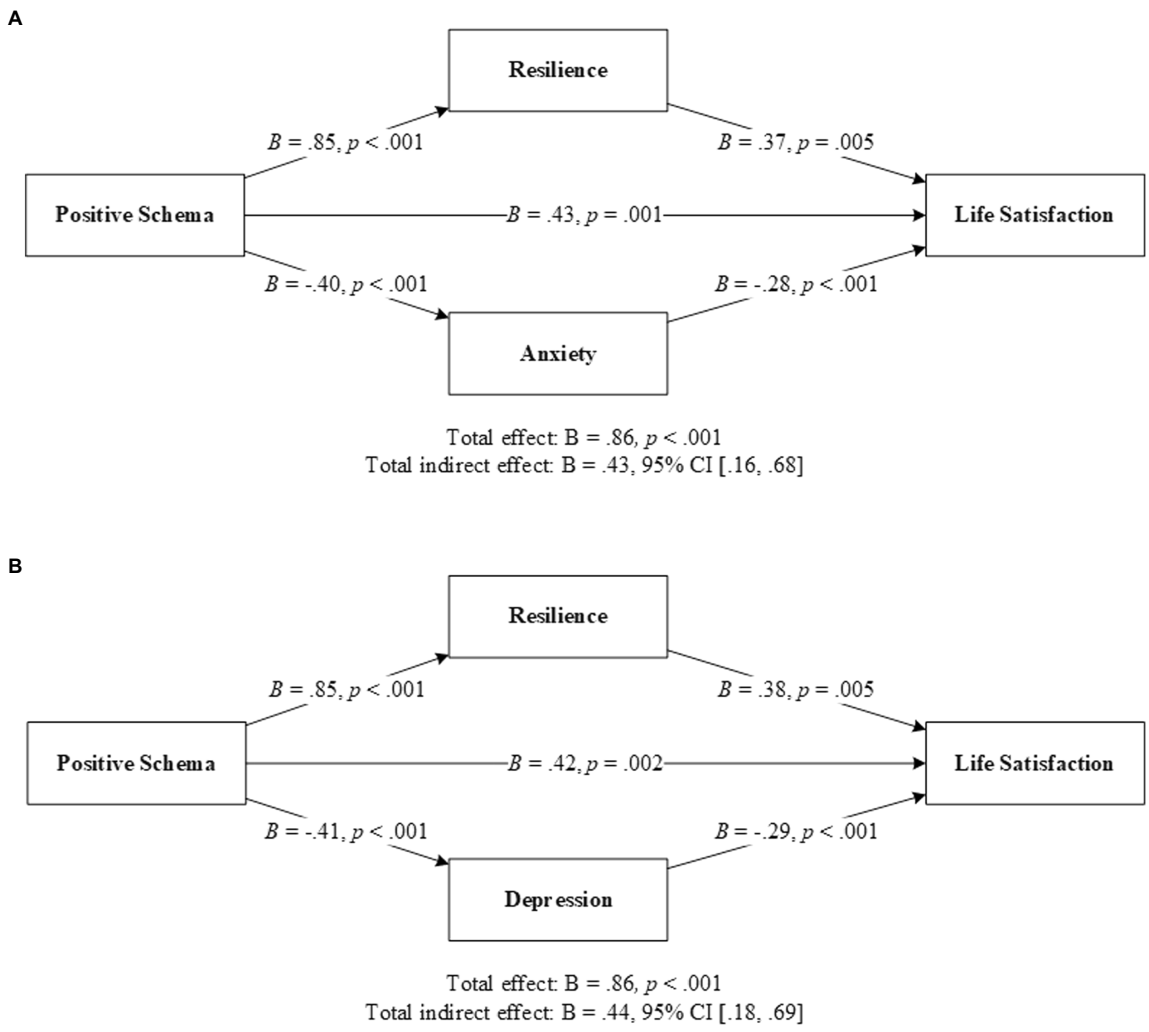
Resilience and anxiety **(A)**/depression **(B)** as parallel mediators between positive schema and life satisfaction. *N* = 129. CI, confidence interval. Solid lines indicate significant paths, and dotted lines indicate nonsignificant paths. Results were based on 5,000 bootstrap samples.

It was possible that resilience and anxiety/depression acted as parallel mediators in the relationship between positive schemas and life satisfaction. Further explorations were conducted to investigate this possibility. The results supported resilience and anxiety/depression as parallel mediators between positive schemas and life satisfaction, as the specific indirect effect of positive schemas through resilience or anxiety/depression was significant (Figure 2; Supplementary Table S2).

## Discussion

This study found that higher levels of positive schemas were associated with greater resilience and life satisfaction, and lower levels of anxiety and depression. This finding is consistent with those of previous studies in the general populations (Keyfitz et al., 2013; Louis et al., 2018; Paetsch et al., 2020; Chi et al., 2022). This study confirmed these relationships in psychiatric inpatients diagnosed with MDD or GAD.

Moreover, this study identified three pathways from positive schemas to life satisfaction: positive schemas → life satisfaction; positive schemas → resilience → life satisfaction; and positive schemas → anxiety/depression → life satisfaction. This suggests that inpatients with higher levels of positive schemas were also more resilient and perceived greater life satisfaction, and this association was partially mediated by lower levels of distress or higher levels of resilience. The direct effect of positive schema and life satisfaction indicates a more fundamental influence that might exist independent of resilience and distress.

In this study, resilience and anxiety/depression were hypothesized as serial mediators between positive schemas and life satisfaction. However, inconsistent with Hypotheses 1 and 2, resilience and anxiety/depression were parallel mediators between positive schemas and life satisfaction. It seems that the indirect effects of positive schemas on life satisfaction were delivered through a dual parallel pathway: increased resilience and decreased distress. Negative emotions mediate the indirect effect of positive schemas on life satisfaction without the involvement of resilience as the former mediator. It is possible that patients whose early core emotional needs were met, perceived fewer negative emotions and greater life satisfaction in a more direct and fundamental way, independent of the involvement of resilience. However, because studies on positive schemas are limited, no comparisons could be made. Replications are required for further verification.

### Implications and limitations

Few studies have examined the relationship between positive schema and life satisfaction, and its potential mechanism in psychiatric patients. The findings of this study improve the understanding of this relatively new concept and theory of positive schemas by identifying the multiple mechanisms through which a positive schema is associated with life satisfaction. This study revealed the direct effect of positive schemas on life satisfaction, independent of distress and resilience. Given that positive schemas are associated with more optimal and less psychopathological outcomes, psychotherapists might integrate promoting positive schemas into their clinical work. Future studies, especially longitudinal ones in clinical settings, are required to explore this possibility and to provide more robust empirical evidence.

This study has some limitations. As it was cross-sectional, the causality or directions of the relationships could not be determined. The participants in this study were inpatients with depression and anxiety, and thus, the findings cannot be generalized to all clinical populations. In addition, this study may be prone to self-selection bias as it was based on retrospective self-reported data and no objective indices were collected.

## Conclusion

This study examined the association between positive schemas and life satisfaction and identified the mechanisms underlying this association in psychiatric inpatients. The findings indicated that inpatients with higher levels of positive schemas were more likely to perceive greater life satisfaction, and this association was partially and parallelly mediated by resilience and distress. Replications, especially longitudinal ones, will help confirm these findings and clarify the directions of the relationships. As promoting mental health not only refers to reducing psychopathologies but also improving positive psychological resources. Enhancing and developing positive schemas may act as positive psychological resources and buffer against future psychopathological outcomes and promote better prognosis. Future studies need to investigate whether the incorporation of promoting EASs into schema therapies can improve individuals’ well-being.

## Data availability statement

The raw data supporting the conclusions of this article will be made available by the authors, without undue reservation.

## Ethics statement

The studies involving human participants were reviewed and approved by the institutional review board of Ningbo Kangning Hospital (NBKNYY-2021-LC-50). The patients/participants provided their written informed consent to participate in this study.

## Author contributions

DC and YZ: conceptualization and writing - review and editing. YW and XD: methodology. HZ, YW, and HM: formal analysis and investigation. HZ, HM, and XD: writing - original draft preparation. DC: funding acquisition. YZ: supervision. All authors contributed to the article and approved the submitted version.

## Funding

The research was funded by Ningbo Medical and Health Brand Discipline (PPXK2018-08).

## Conflict of interest

The authors declare that the research was conducted in the absence of any commercial or financial relationships that could be construed as a potential conflict of interest.

## Publisher’s note

All claims expressed in this article are solely those of the authors and do not necessarily represent those of their affiliated organizations, or those of the publisher, the editors and the reviewers. Any product that may be evaluated in this article, or claim that may be made by its manufacturer, is not guaranteed or endorsed by the publisher.
