# Supplementary material for: Relationships between positive schemas and life satisfaction in psychiatric inpatients
Source: Front Psychol. 2022 Dec 15;13:1061516. doi: 10.3389/fpsyg.2022.1061516 (PMC9798195; doi:10.3389/fpsyg.2022.1061516)
Supplement: Supplementary file 1 [file Table_1.pdf]

**Supplementary Table 1**

*Results of Linear Regression Models Used to Examine the Direct Effect of Positive Schema and Serial Mediation of Resilience and Anxiety/Depression on Life Satisfaction*

| Predictor | *B* | *SE* | *t* | 95% CI | *p* |
|---|---|---|---|---|---|
| Age | .004 | .005 | .78 | [-.006, .01] | .43 |
| Direct | | | | | |
| Positive schema total | **.43** | .13 | 3.38 | [.18, .68] | .001 |
| Indirect | | | | | |
| Resilience | **.31** | .13 | | [.05, .56] | |
| Resilience and Anxiety | .04 | .06 | | [-.05, .18] | |
| Anxiety | .07 | .05 | | [-.02, .18] | |
| Total indirect effect | **.43** | .13 | | [.18, .67] | |
| Total effect | **.86** | .07 | 12.19 | [.72, 1.00] | < .001 |
| $R = .81$, $R^2 = .66$., $F$ (4, 124) = 60.17, $p < .001$ | | | | | |
| Age | .006 | .005 | 1.15 | [-.004, .02] | .25 |
| Direct | | | | | |
| Positive schema total | **.42** | .13 | 3.25 | [.16, .67] | .002 |
| Indirect | | | | | |
| Resilience | **.32** | .13 | | [.04, .57] | |
| Resilience and Depression | .06 | .06 | | [-.04, .19] | |
| Depression | .06 | .05 | | [-.02, .18] | |
| Total indirect effect | **.44** | .13 | | [.18, .68] | |
| Total effect | **.86** | .07 | 12.19 | [.72, .97] | < .001 |
| $R = .81$, $R^2 = .66$, $F$ (4, 124) = 59.67, $p < .001$ | | | | | |

*Note.* $N = 129$. Results were based on 5000 bootstrap samples. CI = confidence interval.
