# Supplementary material for: Relationships between positive schemas and life satisfaction in psychiatric inpatients
Source: Front Psychol. 2022 Dec 15;13:1061516. doi: 10.3389/fpsyg.2022.1061516 (PMC9798195; doi:10.3389/fpsyg.2022.1061516)
Supplement: Supplementary file 2 [file Table_2.pdf]

Supplementary Table 2

Results of Linear Regression Models Used to Examine the Direct Effect of Positive Schema and Parallel Mediation of Resilience and Anxiety/Depression on Life Satisfaction

Predictor	*B*	*SE*	*t*	95% CI	*p*
Age	.001	.005	.86	[-.01, .01]	.86
Direct					
Positive schema total	**.43**	.13	3.38	[.18, .68]	.001
Indirect					
Resilience	**.31**	.13		[.04, .57]	
Anxiety	**.11**	.05		[.03, .23]	
Total indirect effect	**.43**	.13		[.16, .68]	
Total effect	**.86**	.07	12.19	[.72, 1.00]	< .001
R = .81, R^2 = .66., F (4, 124) = 60.17, $p < .001$					
Age	.006	.005	1.13	[-.004, .02]	.26
Direct					
Positive schema total	**.42**	.13	3.25	[.16, .67]	.002
Indirect					
Resilience	**.32**	.13		[.06, .58]	
Depression	**.12**	.05		[.04, .23]	
Total indirect effect	**.44**	.13		[.18, .69]	
Total effect	**.86**	.07	12.19	[.72, 1.00]	< .001
R = .81, R^2 = .66, F (4, 124) = 59.67, $p < .001$					

Note. N = 129. Results were based on 5000 bootstrap samples. CI = confidence interval.
